# Neural stem cell-conditioned medium ameliorates Aβ_25–35_-induced damage in SH-SY5Y cells by protecting mitochondrial function

**DOI:** 10.17305/bjbms.2020.4570

**Published:** 2021-04

**Authors:** Guoyong Jia, Zengyan Diao, Ying Liu, Congcong Sun, Cuilan Wang

**Affiliations:** Department of Neurology, Qilu Hospital, Shandong University, Jinan, China

**Keywords:** Alzheimer’s disease, neural stem cell-conditioned medium, Aβ_25–35_, mitochondria, apoptosis

## Abstract

Inhibition of amyloid β (Aβ)-induced mitochondrial damage is considered crucial for reducing the pathological damage in Alzheimer’s disease (AD). We evaluated the effect of neural stem cell-conditioned medium (NSC-CDM) on Aβ_25–35_-induced damage in SH-SY5Y cells. An *in vitro* model of AD was established by treating SH-SY5Y cells with 40 μM Aβ_25–35_ for 24 h. SH-SY5Y cells were divided into control, Aβ_25–35_ (40 μM), Aβ_25–35_ (40 μM) + NSC-CDM, and Aβ_25–35_ (40 μM) + neural stem cell-complete medium (NSC-CPM) groups. Cell viability was detected by CCK-8 assay. Apoptosis, reactive oxygen species (ROS) production, and mitochondrial membrane potential (MMP) were detected by flow cytometry. Malondialdehyde content was detected by ELISA assay. Western blot analysis was used to detect cytochrome c release and apoptosis-related proteins. Transmission electron microscopy was used to observe mitochondrial morphology. Cell viability significantly decreased and apoptosis significantly increased in SH-SY5Y cells treated with Aβ_25–35_, and both effects were rescued by NSC-CDM. In addition, NSC-CDM reduced ROS production and significantly inhibited the reduction of MMP caused by Aβ_25–35_. Furthermore, NSC-CDM ameliorated Aβ_25–35_-induced reduction in Bcl-2 expression levels and increased the expression levels of cytochrome c, caspase-9, caspase-3, and Bax. Moreover, Aβ_25–35_ induced the destruction of mitochondrial ultrastructure and this effect was reversed by NSC-CDM. Collectively, our findings demonstrated the protective effect of NCS-CDM against Aβ_25–35_-induced SH-SY5Y cell damage and clarified the mechanism of action of Aβ_25–35_ in terms of mitochondrial maintenance and mitochondria-associated apoptosis signaling pathways, thus providing a theoretical basis for the development of novel anti-AD treatments.

## INTRODUCTION

Alzheimer’s disease (AD) is the most common neurodegenerative disease. Recently, AD has attracted increasing attention for its social harm and high incidence in the elderly population [1]. With the global aging of the population, the development of prevention strategies and effective treatments for AD is considered extremely urgent. The pathogenesis of AD is complex, with the development of senile plaques and neurofibrillary tangles formed by β-amyloid (amyloid β, Aβ) deposition considered hallmark features [2]. Studies have shown that Aβ can directly or indirectly damage the structure and function of mitochondria, resulting in the induction of oxidative stress and the activation of the apoptotic signaling pathway cascade [3]. Moreover, these processes in themselves serve to promote Aβ production, further aggravating mitochondrial damage and resulting in a vicious cycle, leading to neuronal degeneration and apoptosis. Therefore, inhibition of Aβ-induced mitochondrial damage is considered crucial for reducing the pathological damage in AD [4].

Neural stem cells (NSCs) are a type of stem cell with self-renewal and neural differentiation capability. NSCs are abundant in the subventricular zone of adults and the subgranular layer of the hippocampal dentate gyrus [5], which serves as a stem cell pool for biologically replacing damaged nerve tissue. The unique neurorestorative capacity of the hippocampal dentate gyrus makes NSC transplantation the most promising treatment for a variety of neurological diseases, including Parkinson’s disease and AD [6]. To date, NSC transplantation methods have achieved good therapeutic effects in basic experiments. However, the isolation of NSCs is difficult and ethical issues exist surrounding their use. In addition, exogenous cell transplantation often adversely affects the growth of the transplanted cells due to host immune rejection and damage to the pathological microenvironment [7]. Therefore, the clinical use of NSCs in transplantation procedures faces a number of obstacles [8]. Considering that the repair mechanism of NSC ­transplantation involves the replacement of the original neural tissue, there are additional requirements to consider, including immune conditioning and neurotrophic support produced by associated paracrine products [9]. Studies have shown that NSC-conditioned medium (NSC-CDM) can increase the *in vitro* expression of M2 macrophages, reduce M1 type activation, and inhibit the release of multiple inflammatory factors [10]. Similarly, *in vivo* experiments have shown that the injection of NSC-CDM into rats with spinal cord injury increases the bridging needed between the corticospinal tract and interneurons, thus reducing neuronal apoptosis and promoting motor function recovery [11]. Therefore, the use of NSC-CDM to replace the original secretions of these cells has become a new therapeutic strategy that can effectively avoid a number of problems, including ethics issues, transplant cell survival, cell preservation, and transportation.

In this study, our findings demonstrated that NSC-CDM is protective against Aβ_25–35_-induced cytotoxicity, including apoptosis, reduced cell viability, and damage to the mitochondrial ultrastructure, in SH-SY5Y cells. In addition, further analysis of mitochondrial apoptosis-related proteins indicated that the protective effect of NSC-CDM is due to the modulation of the intrinsic apoptotic pathway.

## MATERIALS AND METHODS

### Aβ_25–35_ preparation

Five milligrams of Aβ_25–35_ (Sigma-Aldrich, St. Louis, MO, USA) was dissolved in 5 mL double-distilled water. A micron microporous filter (0.22 μm) was sterilized by filtration under sterile conditions and placed in a 37°C incubator for 7 days. A small sample was taken for protein concentration determination and stored at -20°C for later use.

### Cell culture and treatment

Logarithmic growth phase human SH-SY5Y cells (N7800-100, Thermo Fisher Scientific, USA) were collected, counted, and resuspended in Dulbecco’s Modified Eagle Medium/Ham’s F–12 (DMEM/F-12) complete medium [CPM] (11320033, Gibco, USA) containing 10% fetal bovine serum [FBS] (10099133, Gibco) and 1% double antibody. The cell concentration was adjusted to 1 × 10^5^ cells/mL and the cells were seeded in 6-well plates, with 2 mL of cell suspension per well. The plates were incubated at 37°C overnight at 5% CO_2_. After the cells were fully attached, the medium in the wells was discarded and the plates were prepared according to the experimental group. For the control group, 2 mL of DMEM/F-12 medium containing 10% FBS was added to the 6-well plate. For the Aβ_25–35_ group, Aβ_25–35_ and DMEM/F-12 medium containing 10% FBS were added to the 6-well plate, with the final concentration of Aβ_25–35_ 40 μM. For the Aβ_25–35_ + NSC-CDM group, Aβ_25–35_ and 10% FBS containing NSC-CDM were added to the 6-well plate, with the final concentration of Aβ_25–35_ 40 μM. For the Aβ_25–35_ + NSC-CPM group, Aβ_25–35_ and 10% FBS containing NSC-CPM were added to the 6-well plate, with the final concentration of Aβ_25–35_ 40 μM. The isolation and culturing of the NSCs and the NSC-CDM were performed according to our previous study [12].

### CCK-8 analysis

SH-SY5Y cells were grown at 2–4 × 10^4^ cells/well in 96-well microplates. The CCK-8 solution (CK04, Sigma-Aldrich, USA) was then added to the medium to a final concentration of 0.5 mg/mL and incubated for 4 h at 37°C. The absorbance was read at 450 nm by Multiskan FC (Thermo Scientific, USA) and the cell viability was determined.

### Apoptosis analysis

Using an *in situ* cell death detection kit (Roche, Mannheim, Germany), the cells were grown on coverslips, followed by the terminal deoxynucleotidyl transferase-mediated dUTP nick end labeling (TUNEL) assay. After TUNEL labeling, the sections were observed using a light microscope (Olympus, Tokyo, Japan) to detect apoptotic cells at ×400 magnification, with a view size area of 0.344 mm^2^. The cells that were positively stained with the TUNEL stain presented as a dark red color under the light microscope and were considered to be apoptotic.

### Flow cytometry analysis

The Annexin V-FITC/PI Apoptosis Detection Kit (Becton Dickinson, Rutherford, NJ, USA) was used for the quantification of cellular apoptosis. Briefly, the cells were resuspended in 200 μL annexin binding buffer containing 5 μL PI and 10 μL annexin V-FITC in the dark for 10 min at 25°C. Flow cytometry (Abcam, USA) was used to analyze the double-stained cells.

### Assessment of reactive oxygen species (ROS) production

Mitochondrial ROS production was evaluated using specific ROS kits (GenMed Scientifics Inc., Wilmington, DE). Mitochondrial fractions (50 μg) were cultured with 6-chloromethyl-2’,7’-dichlorodihydro-fluorescein diacetate (CM-H2DCFDA) at 37°C for 15 min. Fluorescence, with excitation and emission wavelengths of 490 and 530 nm, respectively, was monitored by a fluorescence spectrophotometer.

### Determination of malondialdehyde (MDA) activity

SH-SY5Y cells were centrifuged for 15 min at 4500 rpm. The samples were stored at −80°C prior to the analysis of MDA activity, which was evaluated using a commercial ELISA kit (A003-4-4, Nanjing Built Bio, Nanjing, China).

### Transmission electron microscopy (TEM)

SH-SY5Y cells at 5 × 10^6^ cells/mL were incubated in Schneider medium at 25°C for 24 h. After washing with phosphate-buffered saline (PBS), the cells were fixed in 2.5% glutaraldehyde in 0.1 M sodium cacodylate buffer (pH 7.2) at 25°C for 40 min and post-fixed in a solution containing 1% osmium tetroxide, 0.8% potassium ferricyanide, and 2.5 mM CaCl_2_ for 20 min. The cells were then dehydrated with acetone and embedded in PolyBed 812 resin. Ultrathin sections (0.06 mm) were sliced and stained with uranyl acetate and lead citrate, followed by examination with a JEM-1200 EX electron microscope by a blindedexaminer.

### Determination of mitochondrial membrane potential (MMP)

MMP was evaluated using a specific MMP kit (Beyotime, Haimen, China) that included JC-1 (5,5’,6,6’-tetrachloro-1,1’,3,3’-tetraethyl-imida carbocyanine iodide, C2006, Biyuntian, Shanghai, China), which is a fluorochrome that becomes incorporated into cells depending on the status of the MMP. In this process, staining for reduced JC-1, which emits green fluorescence, indicates a disruption of the mitochondrial inner-membrane potential. Briefly, SH-SY5Y cells in 6-well plates were processed as described in previous experiments, washed with PBS, and cultured with the JC-1 solution at 37°C for 20 min in the dark. The cells were then washed twice with PBS and resuspended in PBS (500 μL). Fluorescence was evaluated by a BD FACSAria II flow cytometer system (BD, Franklin Lakes, NJ). The results are shown in terms of the proportion of cells with a low MMP.

### Western blot analysis

Proteins were examined via Western blot analysis using monoclonal antibodies against the cytochrome c (1:1000, Ab13575, Abcam, USA), caspase-9 (1:1000, Ab52298, Abcam), caspase-3 (1:500, Ab2302, Abcam), Bcl-2-associated X protein [Bax] (1:2000, Ab32503, Abcam), and B-cell lymphoma 2 [Bcl-2] (1:500, Ab32124, Abcam, USA) proteins. β-actin (1:5000, Sigma, USA) served as the loading control. A horseradish peroxidase (HRP)-labeled secondary antibody (1:1000, Santa Cruz, USA) was used and cultured with the cells for 1 h at 25°C. Quantification of the band density was performed using a LI-COR Odyssey infrared imaging system (LI-COR Bio-science, Nebraska, USA).

### Statistical analysis

The GraphPad Prism version 8.00 for Windows (GraphPad Software, La Jolla California USA) was used to analyze the data. All experiments were repeated 3 times and the mean ± standard deviation was used for all analyses. ANOVA was used to determine whether marked differences existed among the experimental groups, with *p* < 0.05 regarded as significant.

## RESULTS

### Aβ_25–35_-induced damage in SH-SY5Y cells

**FIGURE 1:**
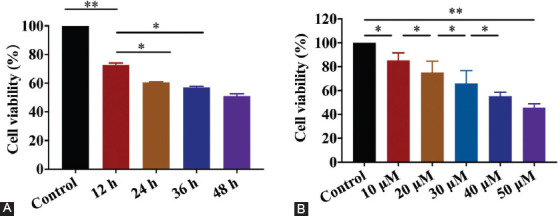
Different concentration and time of Aβ_25–35_ treatment in SH-SY5Y cells. (A) CCK-8 detection of Aβ_25–35_ (30 μM) treatment in SH-SY5Y cells for 12, 24, 36, and 48 h. (B) CCK-8 detection of Aβ_25–35_ (0, 10, 20, 30, 40, and 50 mM) treatment in SH-SY5Y cells for 24 h. *p < 0.05, **p < 0.01. When the time was increased to 36 h, the cell viability decreased to 58.62 ± 1.26% compared with the control group. Different concentrations of Aβ_25–35_ were then used to treat SH-SY5Y cells for 24 h. As the concentration of Aβ_25–35_ increased, the cell survival rate decreased gradually. At 40 μM, the cell viability decreased to 56.62 ± 1.26% compared with the control group.

Aβ_25–35_ (40 μM) was used to treat the SH-SY5Y cells for different time periods and the cell survival rate was shown to decrease with time. When the time was increased to 36 h, the cell viability decreased to 58.62 ± 1.26% compared with the control group (*p* < 0.05; Figure 1A). Different concentrations of Aβ_25–35_ were then used to treat the SH-SY5Y cells for 24 h. As the concentration of Aβ_25–35_ increased, the cell survival rate decreased gradually. At 40 mM, the cell viability decreased to 56.62 ± 1.26% compared with the control group (*p* < 0.05; Figure 1B).

### NSC-CDM rescued Aβ_25–35_-induced cytotoxicity, including decreased cell viability and increased apoptosis, in SH-SY5Y cells

**FIGURE 2:**
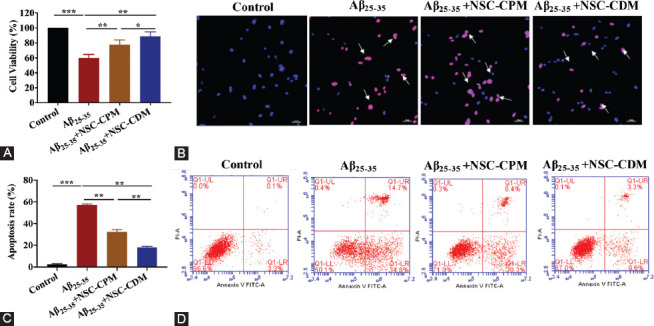
Neural stem cell-conditioned medium (NSC-CDM) protected against Aβ_25–35_-induced toxicity, including decreased cell viability and increased apoptosis, in SH-SY5Y cells. (A) The cell viability of SH-SY5Y cells was evaluated by CCK-8 assay in the control, Aβ_25–35_ (40 μM), Aβ_25–35_ (40 μM) + neural stem cell-complete medium (NSC-CPM), and Aβ_25–35_ (40 μM) + NSC-CDM groups for 24 h. (B and C) The apoptotic rates of the cells were labeled with the TUNEL assay in the control, Aβ_25–35_ (40 μM), Aβ_25–35_ (40 μM) + NSC-CPM, and Aβ_25–35_ (40 μM) + NSC-CDM groups for 24 h. The white arrow represents apoptotic cells. Scale bar = 100 mm. (D) Apoptotic cells were examined by annexin V-FITC/PI double-staining in the control, Aβ_25–35_ (40 μM), Aβ_25–35_ (40 μM) + NSC-CPM, and Aβ_25–35_ (40 μM) + NSC-CDM groups for 24 h. *p < 0.05, **p < 0.01, ***p < 0.001. Aβ_25–35_ significantly decreased the cell viability of SH-SY5Y cells as compared with the control group. In contrast, both NSC-CPM and NSC-CDM had an inhibitory effect on the cytotoxicity induced by Aβ_25–35_, with the NSC-CDM group demonstrating a higher cell viability than the NSC-CPM group. Next, we applied TUNEL and Annexin V-FITC/PI double-staining to determine the number of apoptotic SH-SY5Y cells. The nuclear fragmentation that is a characteristic feature of apoptotic cells was clearly observed in the Aβ_25–35_-induced SH-SY5Y cells. In addition, condensed nuclei were also identified. However, the numbers of TUNEL-positive nuclei were significantly lower in the NSC-CPM or NSC-CDM + Aβ_25–35_-treated cells, with the NSC-CDM group demonstrating a lower number of TUNEL-positive nuclei than the NSC- CPM group. In addition, cellular apoptosis was examined by Annexin V-FITC/PI double-staining and showed the same trends.

To investigate the effects of NSC-CDM in SH-SY5Y cells, the cell viability of the cells was evaluated by CCK-8 assay in the control, Aβ_25–35_ (40 μM), Aβ_25–35_ (40 μM) + NSC-CPM, and Aβ_25–35_ (40 μM) + NSC-CDM groups for 24 h. As shown in Figure 2A, Aβ_25–35_ significantly decreased the cell viability of the SH-SY5Y cells as compared with the control group (*p* < 0.001). In contrast, both NSC-CPM and NSC-CDM had an inhibitory effect on the cytotoxicity induced by Aβ_25–35_ (*p* < 0.001 and *p* < 0.01, respectively), with the NSC-CDM group demonstrating a higher cell viability than the NSC-CPM group (*p* < 0.05). Next, we applied TUNEL and annexin V-FITC/PI double-staining to determine the number of apoptotic SH-SY5Y cells. As demonstrated in Figure 2B and C, the nuclear fragmentation that is a characteristic feature of apoptotic cells was clearly observed in the Aβ_25–35_-induced SH-SY5Y cells. In addition, condensed nuclei were also identified. However, the numbers of TUNEL-positive nuclei were significantly lower in the NSC-CPM or NSC-CDM+ Aβ_25–35_-treated cells, with the NSC-CDM group demonstrating a lower number of TUNEL-positive nuclei than the NSC-CPM group. In addition, cellular apoptosis was examined by annexin V-FITC/PI double-staining and showed the same trends (Figure 2D).

### NSC-CDM protected against mitochondrial pathway-related apoptosis induced by Aβ_25–35_ in SH-SY5Y cells

**FIGURE 3:**
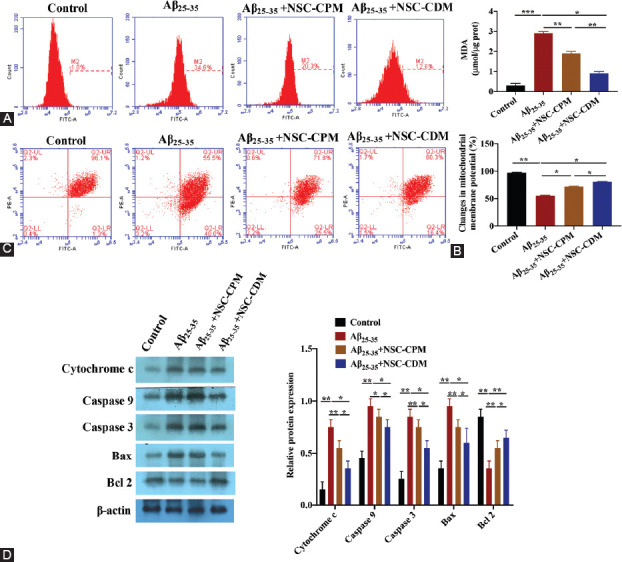
Neural stem cell-conditioned medium (NSC-CDM) protected against mitochondrial pathway-related apoptosis in SH-SY5Y cells induced by Aβ_25–35_. (A) Flow cytometry was used to detect the relative intensity of reactive oxygen species (ROS) in the SH-SY5Y cells in the control, Aβ_25–35_ (40 μM), Aβ_25–35_ (40 μM) + NSC-CPM, and Aβ_25–35_ (40 μM) + NSC-CDM groups for 24 h. (B) The MDA contents of the SH-SY5Y cells were assessed by ELISA kits in the control, Aβ_25–35_ (40 μM), Aβ_25–35_ (40 μM) + NSC-CPM, and Aβ_25–35_ (40 μM) + NSC-CDM groups for 24 h. (C) Changes in the MMP by JC-1 in the control, Aβ_25–35_ (40 μM), Aβ_25–35_ (40 μM) + NSC-CPM, and Aβ_25–35_ (40 μM) + NSC-CDM groups for 24 h. (D) Cytochrome c, caspase-9, caspase-3, Bcl-2-associated X protein (Bax), and B-cell lymphoma 2 (Bcl-2) protein expressions were evaluated by Western blot analysis in the control, Aβ_25–35_ (40 μM), Aβ_25–35_ (40 μM) + NSC-CPM, and Aβ_25–35_ (40 μM) + NSC-CDM groups for 24 h. *p < 0.05, **p < 0.01, ***p < 0.001. The ROS content of the Aβ_25–35_ group was significantly higher than that of the control group. The ROS content in the NSC-CPM and NSC-CDM+Aβ_25–35_ group was significantly lower than that in the Aβ_25–35_ group. The results of MDA content assays were similar to those of ROS assays. In addition, a decrease in MMP became obvious in the Aβ_25–35_ group. These effects were reversed by the administration of NSC-CPM or NSC-CDM. The results from the Western blot analysis showed a decrease in the Bcl-2 expression level and an increase in the cytochrome c, caspase-9, caspase-3, and Bax expression levels in the Aβ_25–35_ group compared with the control group.

To further clarify how NSC-CDM protected against mitochondrial pathway apoptosis induced by Aβ_25–35_, we detected ROS, MDA, MMP, and mitochondrial pathway apoptosis-related proteins in the control, Aβ_25–35_ (40 μM), Aβ_25–35_ (40 μM) + NSC-CPM, and Aβ_25–35_ (40 μM) + NSC-CDM groups. ROS assays showed that the ROS content of the Aβ_25–35_ group was significantly higher than that of the control group (*p* < 0.001). The ROS contents in the NSC-CPM and NSC-CDM+Aβ_25–35_ groups were significantly lower than that in the Aβ_25–35_ group (*p* < 0.01 and *p* < 0.05, respectively; Figure 3A). The results of the MDA content assays were similar to those of the ROS assays (Figure 3B). In addition, a decrease in the MMP became obvious in the Aβ_25–35_ group. These effects were reversed by the administration of NSC-CPM or NSC-CDM (Figure 3C). To further clarify how NSC-CDM inhibited mitochondrial pathway apoptosis, we used Western blot analysis to measure the expressions of cytochrome c, caspase-9, caspase-3, Bax, and Bcl-2 in the control, Aβ_25–35_ (40 μM), Aβ_25–35_ (40 μM) + NSC-CPM, and Aβ_25–35_ (40 μM) + NSC-CDM groups. As illustrated in Figure 3D, the results from the Western blot analysis showed a decrease in the Bcl-2 expression level and an increase in the cytochrome c, caspase-9, caspase-3, and Bax expression levels in the Aβ_25–35_ group compared with the control group (all *p* < 0.01). These effects were further ameliorated by the administration of NSC-CPM or NSC-CDM.

### NSC-CDM protected against Aβ_25–35_-induced mitochondrial ultrastructure damage in SH-SY5Y cells

**FIGURE 4:**
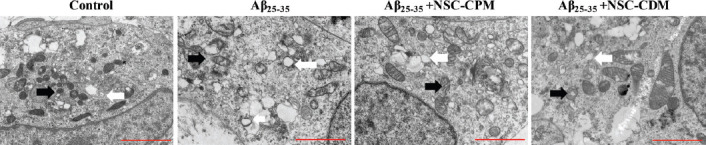
Transmission electron microscopy was used to detect the ultrastructure of the mitochondria in the SH-SY5Y cells in the control, Aβ_25–35_ (40 μM), Aβ_25–35_ (40 μM) + neural stem cell-conditioned medium (NSC-CPM), and Aβ_25–35_ (40 μM) + NSC-CDM groups for 24 h. The black arrow indicates normal mitochondria, whereas the white arrow indicates swollen mitochondria. Scale bar = 2 mm. Mitochondrial swelling was observed in the SH-SY5Y cells treated with Aβ_25–35_. In particular, the crista of the mitochondria was observed to almost disappear or disintegrate. However, in the NSC-CPM and NSC-CDM+Aβ_25–35_ groups, although most of the mitochondria were swollen and disrupted, we observed some normal mitochondria with the crista split. In addition, mitochondrial swelling in the NSC-CDM + Aβ_25–35_ group was considered mild compared with that of the NSC-CPM + Aβ_25–35_ group.

To further clarify whether NSC-CDM had the capacity to protect the ultrastructure of the mitochondria, we used TEM to observe mitochondrial morphology in the control, Aβ_25–35_ (40 μM), Aβ_25–35_ (40 μM) + NSC-CPM, and Aβ_25–35_ (40 μM) + NSC-CDM groups. As illustrated in Figure 4, mitochondrial swelling was observed in the SH-SY5Y cells treated with Aβ_25–35_. In particular, the crista of the mitochondria was observed to almost disappear or disintegrate. However, in the NSC-CPM and NSC-CDM+Aβ_25–35_ groups, although most of the mitochondria were swollen and disrupted, we observed some normal mitochondria with the crista split. In addition, mitochondrial swelling in the NSC-CDM + Aβ_25–35_ group was considered mild compared with that of the NSC-CPM + Aβ_25–35_ group. Therefore, our results indicated the maintenance of the mitochondrial ultrastructure by the administration of NSC-CPM or NSC-CDM.

## DISCUSSION

AD is a neurodegenerative disease associated with aging and is considered to be the most common form of dementia. The causes of AD include the accumulation of Aβ, oxidative stress, inflammation, and a dysfunction in various pathways, including those related to hormones and the mitochondria [13]. In addition, increased proteolytic degradation of amyloid precursor protein (APP) and the aggregation and deposition of Aβ are considered to be two characteristic pathologies in the development and progression of AD [14]. In particular, Aβ_25–35_ is a core toxic fragment of the full-length Aβ peptide [15] that easily penetrates the cell membrane due to its small size. In addition, the toxicity of Aβ_25–35_ is similar to that of Aβ_1–40_ and Aβ_1–42_ [16]. However, Aβ_25–35_ is a particularly difficult-to-treat peptide because it aggregates rapidly and it quickly becomes toxic, whereas full-length Aβ needs to age for more than a week to aggregate [17]. Therefore, Aβ_25–35_ is commonly used in *in vitro* studies to predict the neuroprotective effects of various drugs that modulate Aβ toxicity [18]. In our current study, Aβ_25–35_ (40 μM) decreased the cell viability of SH-SY5Y cells in a time- and concentration-dependent manner.

Previous studies have demonstrated that NSCs can ­promote the recovery of the nervous system through direct action (i.e., neural replacement) [19] and the indirect bystander secretion of brain-derived neurotrophic factor (BDNF) [20], thereby inhibiting the inflammatory process and enhancing internal glial production [21]. However, the original sources and low survival and neuron differentiation rates [22], along with the potential for NSC tumor formation [23], have limited the clinical application of NSCs. Historically, NSC-CDM has always been discarded as waste because NSCs produce potentially harmful substances in the NSC-CDM during cell division *in vitro*. However, in recent years, NSC-CDM has received increasing attention due to the extensive study of the bystander behavior of NSCs *in vivo*, particularly that of the microvesicles released by NSCs. In addition, NSC-CDM has been shown to exert anti-apoptotic effects both *in vitro* [24] and *in vivo* [11]. To circumvent these potential obstacles and find new therapeutic strategies to treat AD, we first explored the effect of NSC-CDM in Aβ_25–35_-induced SH-SY5Y cells and we consider that NSC-CDM may be an effective treatment for AD. Indeed, our data showed that Aβ_25–35_ significantly decreased cell viability and induced apoptosis in the SH-SY5Y cells, whereas NSC-CDM or NSC-CPM had an inhibitory effect on this toxicity when fibrillation of Aβ_25–35_ occurred. However, an *in vitro* model always has limitations; therefore, animal experiments should be considered in the future.

The oxidation of ROS leads to oxidative damage and neuronal cell death and is known to play an important role in the pathogenesis of neurodegenerative diseases. Antioxidants have been proposed to prevent the toxicity caused by Aβ_25–35_ in AD [25]. Previous studies have shown that MDA inhibits mitochondrial complex I- and complex II-linked respiration and reduces MMP, leading to mitochondrial dysfunction [26]. Bax, a homologous protein to Bcl-2, is a pro-apoptotic protein, while Bcl-2 is an anti-apoptotic protein that inhibits apoptosis. Therefore, the respective levels of Bax and Bcl-2 are considered to be directly related to the regulation of apoptosis. In addition, cytochrome c is an essential component of the respiratory chain and plays an important role in redox and energy metabolism, while also being a key component of the mitochondrial initiation of apoptosis [27]. In this process, the Bax protein acts as a component of the ion channel on the mitochondrial membrane and the upregulation of Bax allows for cytochrome c to cross the mitochondrial membrane, thereby activating the initiation of apoptosis through the activation of caspase-9. Further activation of caspase-3 in the apoptotic cascade results in cellular apoptosis [28]. The downregulation of Bcl-2 prevents it from interfering with cytochrome c release, thereby activating the caspase protease activity of upstream apoptotic proteins and promoting cellular apoptosis [29]. In the present study, the ROS contents of the NSC-CDM and NSC-CPM groups were significantly decreased in Aβ_25–35_-induced SH-SY5Y cells. The results of MDA content assays were similar to those of ROS production assays. In addition, the associated decrease in the MMP was reversed by treatment with NSC-CDM or NSC-CPM. Furthermore, the Western blot results showed that the decreased Bcl-2 expression level and the increased expression levels of cytochrome c, caspase-9, caspase-3, and Bax induced by Aβ_25–35_ were ameliorated by NSC-CDM or NSC-CPM.

Autophagy is an important mechanism for self-protection and self-renewal of cells [30]. Oxidative stress leads to the degradation of mitochondria and the generation of abnormal proteins that cannot be degraded by the ubiquitin-proteasome system, thus requiring autophagy [31]. Indeed, the timely degradation of excess, damaged, and aging proteins and organelles prevents oxidative stress cascades, while also providing essential materials for cellular reconstitution, regeneration and repair, thus maintaining cellular homeostasis. Collectively, our findings indicated that Aβ_25–35_ induced damage to the mitochondrial ultrastructure, which was reversed by NSC-CDM or NSC-CPM treatment.

## CONCLUSION

This study demonstrated the protective effect of NCS-CDM on Aβ_25–35_-induced damage in SH-SY5Y cells and clarified the mechanism of action of Aβ_25–35_ in terms of mitochondrial function maintenance and mitochondria-associated apoptosis signaling pathways, thus providing a theoretical basis for the development of novel anti-AD treatments.
